# Morphological Brain Alterations and Network Disorganizations in Individuals With Substance Use Disorders: Differential Effects of Heroin Versus Methamphetamine

**DOI:** 10.1002/brb3.71051

**Published:** 2025-11-14

**Authors:** Xiaoliang Zhou, Wenbin Liang, Mingwu Lou, Jiangpei Zhao

**Affiliations:** ^1^ Department of Radiology Longgang District Central Hospital of Shenzhen Shenzhen China; ^2^ Shenzhen Clinical Medicine College Guangzhou University of Chinese Medicine Shenzhen China; ^3^ Department of Neurology & Biomedical Innovation Center, The Sixth Affiliated Hospital Sun Yat‐sen University Guangzhou China

**Keywords:** cortical thickness, morphological brain networks, network topology, nodal efficiency, substance use disorder, surface‐based morphometry

## Abstract

**Background:**

Heroin and methamphetamine are two widely abused drugs that have profound effects on brain morphology and functioning. This study aims to (1) identify brain structural differences between heroin and methamphetamine users; (2) examine how these drugs differentially affect the topology and functional connectivity of key brain networks; and (3) characterize associations between morphological alterations and clinical symptoms, including anxiety and depression.

**Methods:**

In this study, we collected T1‐weighted magnetic resonance imaging data from 26 heroin‐abstinent (HA) patients, 24 methamphetamine‐abstinent (MA) patients, and 32 healthy controls (HC). All participants were in early abstinence (< 6 months) to minimize acute intoxication and withdrawal confounds while capturing residual brain alterations. Four surface‐based morphological features, including cortical thickness (CT), fractal dimension (FD), gyrification index (GI), and sulcal depth (SD), were analyzed, and morphological brain networks were constructed using Jensen‐Shannon divergence with 210 cortical regions from the Brainnetome Atlas.

**Results:**

Both patient groups showed brain tissue thinning in hearing‐related areas (temporal cortex) and reduced depth in visual processing regions. Heroin users specifically exhibited atrophy in somatosensory cortex regions associated with touch sensation whereas methamphetamine users demonstrated distinctive cortical folding alterations in motor cortex areas related to movement control. Network analysis revealed that heroin users had widespread connection problems affecting brain communication efficiency, while methamphetamine users showed localized damage in specific brain hubs important for memory, attention, and visual processing. Clinical correlations revealed that morphological changes were significantly associated with drug use patterns (frequency and dosage) and psychological symptoms, with anxiety scores negatively correlating with SD in heroin users and depression scores positively correlating with morphological measures in methamphetamine users.

**Conclusions:**

Our findings demonstrate distinct neurobiological signatures of heroin and methamphetamine addiction that persist during early abstinence. Heroin primarily causes widespread network disruption, while methamphetamine leads to focal hub damage. The observed associations between brain morphology and clinical symptoms indicate the practical importance of these structural alterations. These distinct patterns may inform the development of substance‐specific treatment approaches.

## Graphical abstract

Our morphological brain network analysis of 82 participants reveals that these substances leave dramatically different fingerprints that persist even in recovery, with heroin causing global network collapse through increased path lengths across the entire cortex, while methamphetamine selectively damages temporal parietal, and visual processing centers.

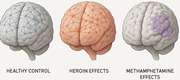

## 1 Introduction

Drug addiction poses significant health risks, including severe neurological and psychological damage, leading to devastating consequences for individuals and society (Volkow and Boyle 2018). Beyond the well‐documented physical harms, chronic use is linked to reductions in subjective well‐being and quality of life (Looby and Earleywine 2007). In China, heroin and methamphetamine are the two most commonly abused substances. Numerous studies have investigated the effects of these drugs on human health from various perspectives, including clinical characteristics, behavioral manifestations, neurocognition, and genetics (Shand et al. 2011; Sun et al. 2021; Thomas et al. 2020; Tian et al. 2022). Recently, an increasing number of studies have focused on the neural mechanisms of heroin and methamphetamine impacts on the human brain using neuroimaging techniques (Gong et al. 2023; Liu et al. 2020; Zilverstand et al. 2018).

Structural magnetic resonance imaging (MRI) is widely used to characterize brain morphology owing to its high test‐retest reliability and excellent spatial resolution. Studies show that drug addiction can lead to morphological alterations in heroin‐abstinent (HA) and methamphetamine‐abstinent (MA) patients (Jan et al. 2012; Wang et al. 2012; Yuan et al. 2009). Reduced gray matter density has been observed in both short‐term and long‐term MA patients, correlating with executive function (Kim et al. 2006). Accumulating evidence shows widespread white‐matter hyperintensities (Bae et al. 2006) and hippocampal volume loss that can be sex‐specific (Du et al. 2015), together with meta‐analytic proof of persisting cognitive deficits in abstinent MA users (Basterfield et al. 2019). Small‐world features and hub reorganization appear to characterize addiction‐related networks (Achard et al. 2006), yet most studies rely on a single morphometric index like gray matter density or cortical thickness (CT), overlooking other morphological characteristics that provide valuable insights into neural mechanisms (Cascino et al. 2020; Hogstrom et al. 2013; Podgórski et al. 2021). Furthermore, recent methodological papers highlight that single‐subject morphological similarity networks can capture individual differences ignored by group‐level analyses (Li et al. 2021; Li et al. 2023; Cai et al. 2023). These frameworks may pave the way toward truly individualized connectomes of brain morphology (Wang and He 2024).

This study seeks to learn about the effects of heroin and methamphetamine addiction on brain morphology and individual morphological brain networks. We employed four surface‐based features (CT, fractal dimension, gyrification index, and sulcul depth (FD, GI, and SD)) to examine regional morphological alterations in HA and MA patients compared to healthy controls (HCs). We also constructed individual morphological brain networks based on these features to assess the global and local disorganizations related to drug use.

## 2 Materials and Methods

### 2.1 Participants

A total of 26 HA and 24 MA patients were recruited from a drug rehabilitation hospital in Shenzhen, China. Thirty‐two HCs were enrolled from the local community, matched by age, education level, and handedness. This study complied with the Declaration of Helsinki and was approved by the Ethics Committee of Longgang Central Hospital. All participants provided informed consent.

#### 2.1.1 Inclusion Criteria for Patients

Participants were required to be diagnosed with a substance use disorder per DSM‐IV criteria and abstinent for less than 6 months. We specifically selected this early abstinence period (< 6 months) to minimize acute intoxication and withdrawal confounds while capturing residual or trait‐like alterations that persist beyond immediate drug effects. This timeframe aligns with prior research in short‐term abstinent cohorts and with evidence demonstrating that neurobiological abnormalities can persist into abstinence, thereby improving both interpretability and participant safety/compliance. Additional inclusion criteria required participants to be aged 18 to 45 years, right‐handed, and have no significant medical history aside from substance use. Handedness was restricted to right‐handed participants to minimize hemispheric laterality confounds, as left‐handedness can be associated with atypical functional and structural brain lateralization patterns.

#### 2.1.2 Exclusion Criteria

Neurological disorders (e.g., epilepsy, stroke, neurodegenerative disease), significant head trauma, major psychiatric conditions other than the target substance use disorders, severe systemic diseases that could affect brain morphology (e.g., uncontrolled hypertension, decompensated hepatic/renal disease), infectious diseases involving the CNS, current psychoactive medications, pregnancy/lactation, and MRI contraindications.

### 2.2 Image Acquisition

MRI data were acquired using a 3T Siemens Prisma with a 64‐channel head coil. T1‐weighted images were collected with the following parameters: repetition time = 2300 ms, echo time = 2.32 ms, field of view = 240 × 240 mm^2^, matrix = 256 × 256, slice thickness = 0.45 mm, with 208 sagittal slices and a voxel size of 0.9 × 0.9 × 0.9 mm^3^.

### 2.3 Structural Image Preprocessing

Structural images were preprocessed using the CAT12 toolbox (http://dbm.neuro.uni‐jena.de/cat12/) within the SPM12 framework. Each image was segmented into gray matter, white matter, and cerebrospinal fluid, followed by bias correction to minimize potential artifacts. Our segmentation pipeline follows the statistical approach originally introduced by Rajapakse et al. (1997) and further refined in CAT12, which yields high agreement with alternative toolkits such as FreeSurfer (Ay et al. 2022). CT was estimated using a projection‐based method (Dahnke et al. 2013); FD, GI, and SD were calculated from spherical harmonic reconstructions (Luders et al. 2006; Yotter et al. 2011). Individual maps were resampled into a common fsaverage template.

### 2.4 Construction of Morphological Brain Networks

Morphological brain networks were constructed using 210 cortical ROIs from the Brainnetome Atlas (Fan et al. 2016) as network nodes. All analyses were conducted using MATLAB with the SPM12 package as the basic platform, with structural image preprocessing performed using the CAT12 toolbox. For network edge definition, the values of all vertices within each ROI were extracted from individual morphological maps (CT, FD, GI, SD) to estimate probability density functions using the ksdensity function in MATLAB. Network edges represented morphological similarity between ROI pairs, quantified using Jensen–Shannon divergence (JSD) applied to the probability distributions of morphometric values within each ROI, with 28 sample points for probability estimation. The notion of linking ROIs via Jensen–Shannon divergence parallels recent minimum‐spanning‐tree work that reduces density bias in network construction (Tewarie et al. 2015).

#### 2.4.1 Network Processing

Networks were thresholded using sparsity‐based criteria (range: 0.03–0.3) and converted to binary form, with minimum spanning tree integration to ensure network connectivity. Small‐world coefficients were normalized (zCp and zLp) by the corresponding average value of 100 matched random networks generated with topological rewiring algorithms preserving identical degree distributions.

#### 2.4.2 Network Properties

Global network measures included clustering coefficient and characteristic path length, both normalized against 100 degree‐matched random networks (zCp, zLp). The nodal network measure was nodal efficiency, calculated for each of the 210 brain regions across all four morphological networks. All topological analyses were performed using the GRETNA toolbox.

### 2.5 Statistical Analysis

Demographic data were analyzed using one‐way ANOVA and chi‐squared tests. Groups were matched for age, education, and handedness (all *p* > 0.05). Gender was not included as a covariate due to the small number of females per group.

Clinical variables, including anxiety and depression symptoms, were assessed using standardized clinical rating scales: the Hamilton Anxiety Rating Scale (HAMA) and Hamilton Depression Rating Scale (HAMD), respectively. Drug abuse patterns, including frequency, duration, and dosage, were collected through structured clinical interviews following standardized protocols.

Between‐group differences were examined using one‐way ANOVA with significance levels estimated through non‐parametric permutation testing (1000 iterations) to avoid distributional assumptions, followed by post‐hoc pairwise comparisons using two‐sample t‐tests when main effects reached significance (*p* < 0.05).

For morphological measure selection in correlation analyses, we employed a data‐driven approach where only brain regions and network measures showing significant between‐group differences (patients vs. healthy controls) in the primary analyses were included in subsequent clinical correlation analyses.

FDR corrections were systematically applied across multiple levels: (1) across all 210 brain regions separately for each morphological index (CT, FD, GI, SD) at the *q* < 0.05 level, (2) across all 210 nodes for network efficiency measures within each network type, and (3) separately within each patient group and clinical variable for correlation analyses to control family‐wise error rates.

Prior to correlation analysis, normality of all clinical variables was systematically assessed using Shapiro–Wilk tests. Results indicated significant departures from normality for HAMA scores (*p* < 0.05), HAMD scores (*p* < 0.05), and drug use variables (all *p* < 0.05). Based on these test results, Spearman rank correlation analyses were employed to examine associations between clinical variables and neuromorphological measures.

## 3 Results

### 3.1 Demographic Characteristics

Demographic features of the three groups are shown in Table 1. The study examined three distinct groups: heroin abstainers (HA, *n* = 26), methamphetamine abstainers (MA, *n* = 24), and healthy controls (HCs, *n* = 32).

**TABLE 1:** Demographic and clinical features of three groups.

Demographic and clinical features	HA (*n* = 26)	MA (*n* = 24)	HCs (*n* = 32)	Statistics	*p*‐value
Gender (F/M)	2/24	5/19	3/29	3.63	0.163
Age (years)	34.1 ± 6.5	33.0 ± 7.0	30.4 ± 8.0	1.844	0.168
Educational duration (years)	8.00 (6.00, 9.00)	9.00 (8.00, 9.00)	8.50 (8.0, 9.3)	5.18	0.075
Duration of abstinence (days)^#^	61.0 (33.0, 91.0)	59.0 (36.0, 79.0)	—	0.00	1.000
Duration of drug abuse (years)^#^	6.0 (4.3, 9.0)	4.5 (2.8, 9.0)	—	−1.20	0.230
Frequency of drug abuse (times/day)^*^	2.0 (1.0, 3.0)	3.0 (2.0, 6.25)	—	−2.88	0.004
Dosage of drug use per time (gram)^*^	0.25 (0.12, 0.50)	0.17 (0.03, 0.26)	—	−1.98	0.047
Dosage of drug use per day (gram)^#^	0.35 (0.20, 0.97)	0.50 (0.20, 0.50)	—	−0.25	0.806
Anxiety score (HAMA)^#^	5.0 (3.0, 11.8)	5.0 (2.8, 9.0)	—	−0.20	0.838
Cigarettes per day^*^	20.0 (10.0, 20.0)	10.0 (5.8, 15.0)	—	−2.94	0.003
Depression score (HAMD)^#^	5.5 (2.0, 8.0)	5.0 (3.0, 10.00)	—	−0.59	0.552

Data are presented as mean ± standard deviation for continuous variables or median (IQR) for non‐normally distributed variables.

Statistical comparisons were performed using chi‐square test (*χ* ^2^) for categorical variables and one‐way ANOVA (F) for continuous variables among the three groups.

**Abbreviations**: F, female; HA, heroin abstinent patients; HCs, healthy controls; M, male; MA, methamphetamine abstinent patients.

Duration of abstinence, duration of drug abuse, frequency and dosage of drug use, cigarettes per day, anxiety score, and depression score were only assessed in patient groups (HA and MA).

^#^ No significant difference between HA and MA groups.

* *p* < 0.05.

The three groups showed no significant differences in demographic characteristics, including age, gender distribution, and education level (all *p* > 0.05). Age distribution was comparable across all groups, with no statistically significant differences observed (*F* = 1.844, *p* = 0.168). The mean age was 34.1 ± 6.5 years for the HA group, 33.0 ± 7.0 years for the MA group, and 30.4 ± 8.0 years for the healthy controls. The HA group consisted predominantly of males (24 males, 2 females), while the MA group showed a more balanced gender distribution (19 males, 5 females), and the healthy control group was also male‐predominant (29 males, 3 females). These non‐significant *p*‐values indicate that the groups were appropriately matched for baseline characteristics, providing a solid foundation for subsequent comparative analyses.

Duration of drug abuse and abstinence was also comparable between HA and MA groups. The HA group showed a mean abstinence duration of 61.0 (33.0, 91.0) days, while the MA group had 59.0 (36.0, 79.0) days. Significant differences in drug use patterns were observed between HA and MA patients. MA patients demonstrated a significantly higher frequency of drug use compared to HA patients (3.0 [2.0, 6.25] versus 2.0 [1.0, 3.0] times/day, *p* = 0.004). Conversely, the dosage consumed per use was significantly lower in the MA group (0.17 [0.03, 0.26] g) compared to the HA group (0.25 [0.12, 0.50] g, *p* = 0.047). Although HA patients showed a longer duration of drug abuse (6.0 [4.3, 9.0] years) compared to MA patients (4.5 [2.8, 9.0] years), this difference did not reach statistical significance (*p* = 0.230). Similarly, no significant difference was found in daily dosage between the two groups (HA: 0.35 [0.20, 0.97] g versus MA: 0.50 [0.20, 0.50] g, *p* = 0.806). These findings reveal distinct drug use behavioral patterns between the groups: MA patients exhibited a “frequent, small‐dose” pattern, while HA patients showed a “less frequent, larger‐dose” pattern. While the overall daily exposure and duration of abuse were comparable between groups, the distinct administration patterns may influence the interpretation of between‐group brain morphological differences.

### 3.2 Abnormal Morphology in Patients

#### 3.2.1 CT

**FIGURE 1:**
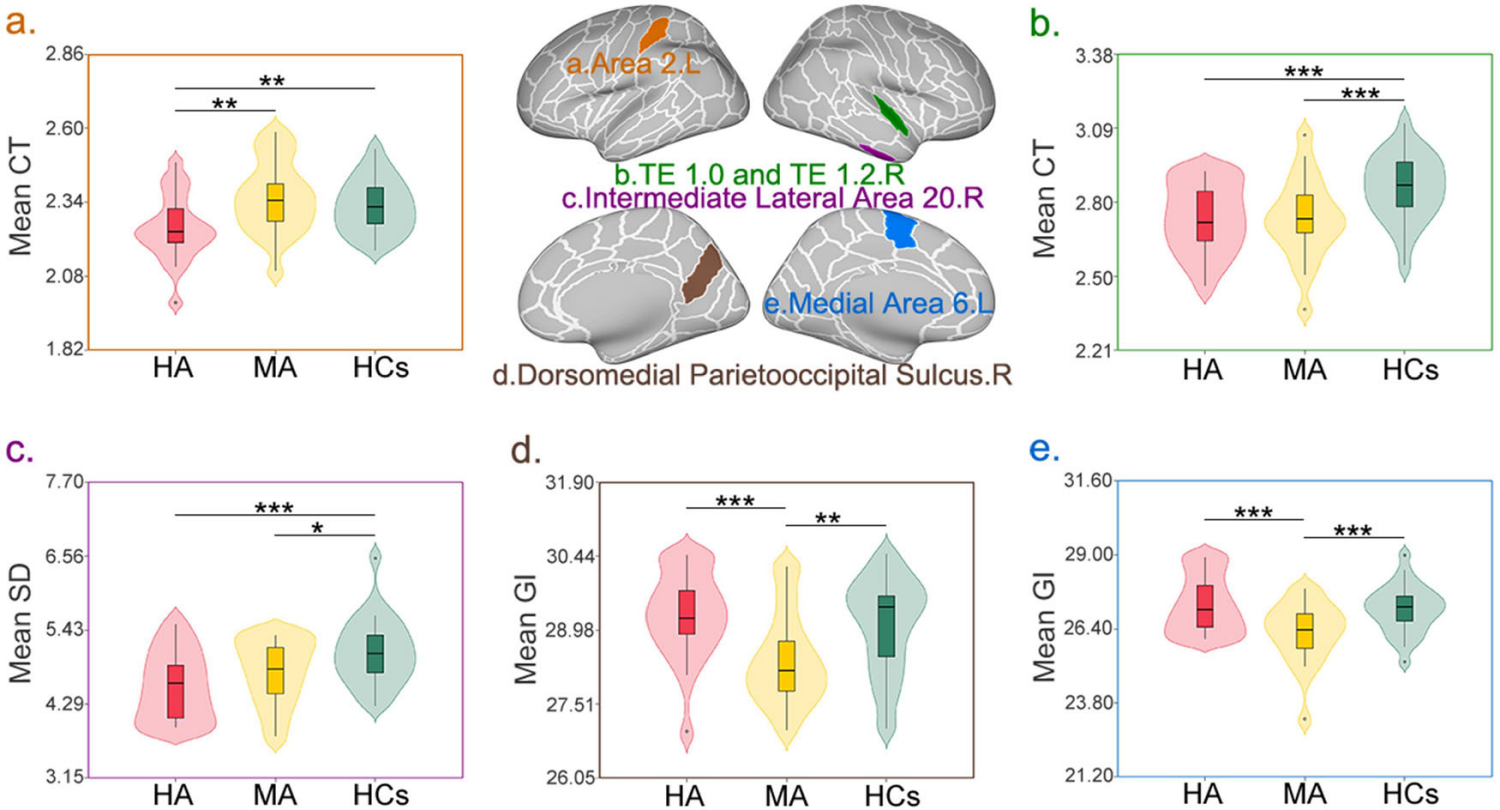
Abnormal cortical morphology in heroin‐ and methamphetamine‐abstinent patients. This figure illustrates regional brain morphological alterations in HA and MA patients compared to HCs. The central brain diagrams show the anatomical locations of significant findings, with each colored region corresponding to the labeled violin plots (**a‐e**). **(a)** Left Area 2 – CT: HA‐specific reduction in cortical thickness. HA patients showed significantly lower CT than both MA patients and HCs (HA < MA and HA < HCs), **(b)** Right TE 1.0 and TE 1.2 – CT: Common reduction in both patient groups. Both HA and MA patients exhibited significantly lower CT than HCs in these primary auditory cortex subregions (HA < HCs and MA < HCs), **(c)** Right Intermediate Lateral Area 20 ‐ SD: Common reduction in both patient groups. Both HA and MA patients showed significantly lower SD than HCs (HA < HCs and MA < HCs), **(d)** Right dorsomedial parieto‐occipital sulcus – GI: MA‐specific reduction in cortical folding. MA patients exhibited significantly lower GI than both HA patients and HCs (MA < HA and MA < HCs), and **(e)** Left Medial Area 6 – GI: MA‐specific reduction in cortical folding. MA patients showed significantly lower GI than both HA patients and HCs (MA < HA and MA < HCs). Statistical significance is indicated by asterisks: *p* < 0.01, * *p* < 0.001. Violin plots display the distribution of values for each group, with median and quartiles shown as box plots within each violin. **Abbreviations**: CT, cortical thickness; GI, gyrification index; HA, heroin‐abstinent; HCs, healthy controls; L, left; MA, methamphetamine‐abstinent; R, right; SD, sulcal depth.

Significant between‐group main effects were observed in the left area 2 and right TE 1.0/TE 1.2 regions. Post‐hoc tests revealed that CT in the left area 2 was significantly lower in HA patients compared to both MA patients (*T* = 3.590, *p* = 0.003) and healthy controls (*T* = 2.788, *p* = 0.008) （Figure 1a）. Similarly, CT in the right TE 1.0 and TE 1.2 regions was significantly reduced in both HA patients (*T* = 3.545, *p* < 0.001) and MA patients (*T* = 3.511, *p* < 0.001) compared to healthy controls (Figure 1b).

#### 3.2.2 FD

No significant between‐group main effects were observed in any brain region for FD measures across the three groups.

#### 3.2.3 GI

Significant between‐group main effects were identified in the left medial area 6 (*F* = 8.256, *p* < 0.001) and right dorsomedial parieto‐occipital sulcus (*F* = 7.728, *p* < 0.001). Post‐hoc analyses revealed that GI in both regions was significantly lower in MA patients compared to both HA patients and HCs (Figure 1d).

#### 3.2.4 SD

A significant between‐group main effect was observed in the right middle lateral area 20 (*F* = 10.517, *p* < 0.001). Post‐hoc tests demonstrated that SD in this region was significantly reduced in both HA patients (*T* = 4.293, *p* < 0.001) and MA patients (*T* = 2.381, *p* = 0.020) compared to healthy controls (Figure 1c).

### 3.3 Global Disorganization of CT Networks in HA Patients

**FIGURE 2:**
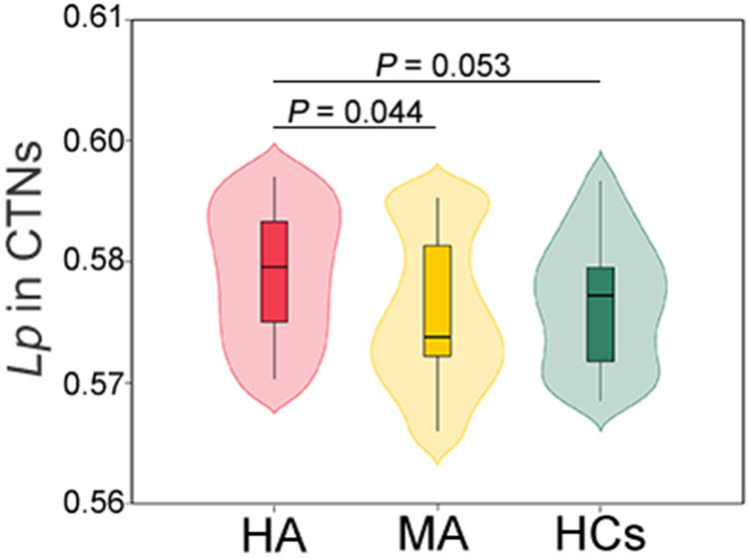
Increased network path length in cortical thickness‐based networks of heroin abstinent patients. Violin plot showing the distribution of Lp values in CTNs across the three groups. The violin shapes display the full distribution of data points, while the inner box plots show the median and interquartile ranges for each group. HA patients exhibited significantly longer Lp compared to MA patients (*p* = 0.044) and marginally longer Lp compared to HCs (*p* = 0.053). These pairwise comparisons were conducted following a marginally significant overall group effect (ANOVA: *F*_2,79_ = 2.872, *p* = 0.058). The longer Lp in HA patients indicate less efficient information transfer within cortical thickness‐based brain networks, suggesting network disorganization specific to heroin abstinence. **Abbreviations**: CTNs, cortical thickness‐based networks; HA, heroin abstinent; HCs, healthy controls; Lp, shortest path length, MA, methamphetamine abstinent,

Among the four morphological networks examined, only the CT network showed marginally significant differences in shortest path length among the three groups (*F* = 2.872, *p* = 0.058). Specifically, HA patients exhibited higher CT network shortest path length compared to both MA patients (*T* = 2.270, *p* = 0.044) and healthy controls (*T* = 1.826, *p* = 0.053), suggesting global network disruption in heroin abstainers (Figure 2).

### 3.4 Local Node Efficiency

Several brain regions showed significant reductions in nodal efficiency, particularly in MA patients:

#### 3.4.1 CT Networks

**FIGURE 3:**
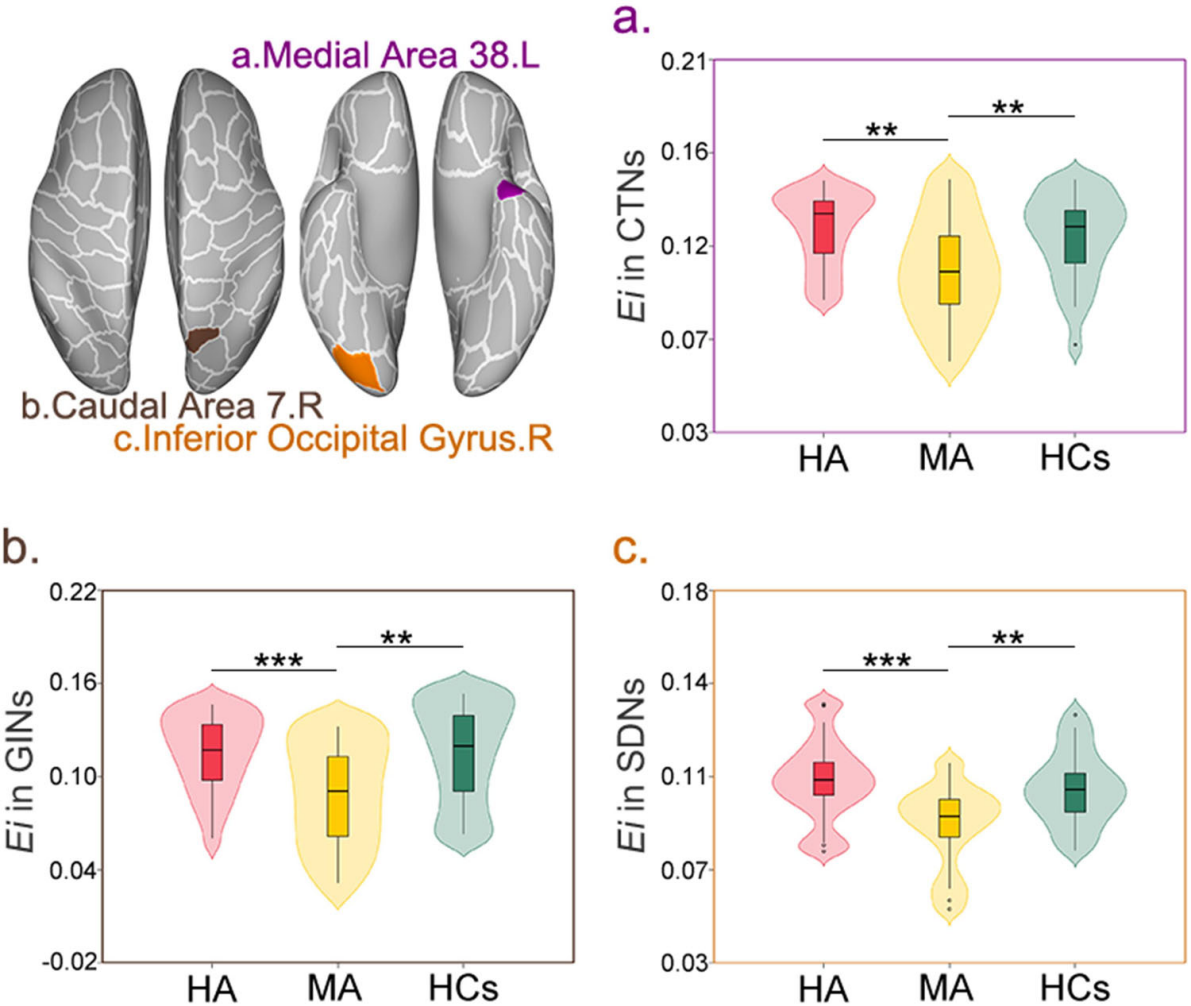
Methamphetamine‐specific disruptions in nodal efficiency across multiple morphological brain networks. This figure demonstrates reduced *E*_i_ in three distinct brain regions among MA patients compared to both HA patients and HCs. The central brain diagrams show the anatomical locations of the three affected regions, with each colored area corresponding to the labeled violin plots below. **(a)** Left Medial Area 38 – CTNs: MA patients exhibited significantly lower *E*_i_ than both HA patients (*p* = 0.002) and HCs (*p* = 0.005) in this temporal lobe region within cortical thickness‐based networks, **(b)** Right Caudal Area 7 – GINs: MA patients showed significantly reduced *E*i compared to HA patients (*p* < 0.001) and HCs (*p* = 0.003) in this parietal region within gyrification index‐based networks, and **(c)** Right Inferior Occipital Gyrus – SDNs: MA patients demonstrated significantly lower Ei than HA patients (**p < 0.001) and HCs (*p* = 0.003) in this occipital region within SDNs. All three regions showed strong overall group effects (all *p* < 0.001), with MA patients consistently exhibiting the lowest nodal efficiency values across different network types. The violin plots display the full distribution of efficiency values, with box plots showing medians and interquartiles. Statistical significance is indicated by asterisks: *p* < 0.01, ***p < 0.001. **Abbreviations**: CTNs, cortical thickness‐based networks; *E*_i_, nodal efficiency; GINs, gyrification index‐based networks; HA, heroin abstinent; HCs, healthy controls; L, left; MA, methamphetamine abstinent; R, right; SDNs, sulcal depth‐based networks.

The left medial area 38 demonstrated significantly reduced nodal efficiency in MA patients (*F* = 7.224, *p* < 0.001) (Figure 3a).

#### 3.4.2 GI Networks

The right caudal area 7 showed significantly decreased nodal efficiency in MA patients (*F* = 8.310, *p* < 0.001) (Figure 3b).

#### 3.4.3 SD Networks

The right inferior occipital gyrus exhibited significantly reduced nodal efficiency in MA patients (*F* = 10.517, *p* < 0.001) (Figure 3c).

### 3.5 Clinical Correlations

To link brain morphological changes to psychological symptoms and drug use patterns, we conducted correlation analyses between neuroimaging measures and clinical variables in both patient groups.

#### 3.5.1 Heroin Abstainers (HA)

**FIGURE 4:**
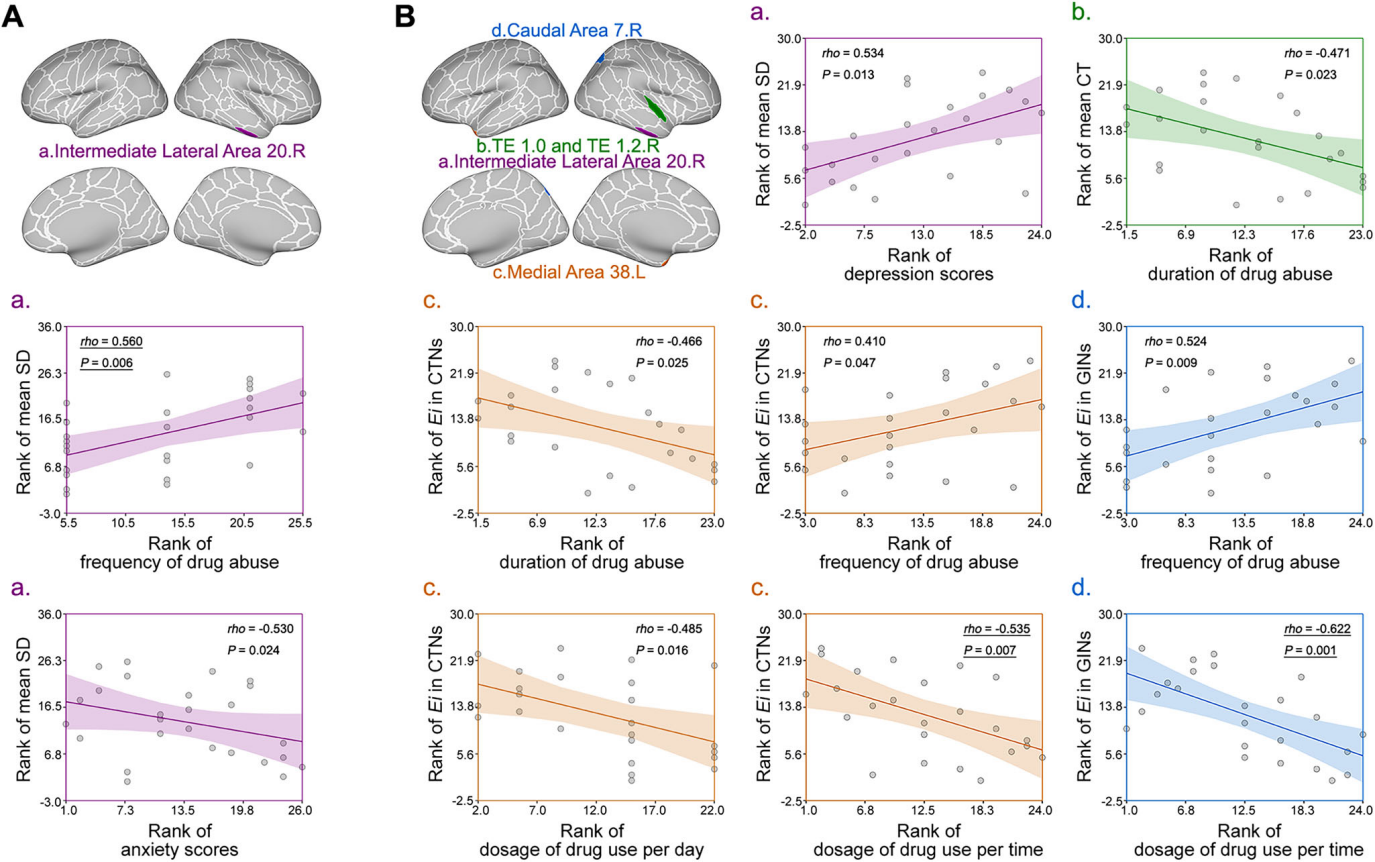
Panel A (HA patients, column 1) displays correlations for HA patients, and Panel B (MA patients, columns 2–4) shows correlations for MA patients. Brain diagrams indicate the anatomical locations of regions showing significant correlations, with colors corresponding to specific regions: **(a)** Right intermediate lateral area 20 (purple), **(b)** Right TE 1.0 and TE 1.2 (green), **(c)** Left medial area 38 (yellow), and **(d)** Right caudal area 7 (blue). The brain diagram shows the right intermediate lateral area 20 (purple). Frequency of heroin use positively correlates with sulcal depth (SD) (rho = 0.560, p = 0.006), while anxiety scores negatively correlate with SD (rho = −0.530, p = 0.024). Abbreviations: CT, cortical thickness; CTNs, cortical thickness‐based networks; Ei, nodal efficiency; GINs, gyrification index‐based networks; HA, heroin‐abstinent; MA, methamphetamine‐abstinent; SD, sulcal depth.

A few significant correlations were identified between clinical variables and brain morphology in HA patients. Drug abuse frequency showed a significant positive correlation with SD in the right middle lateral area 20 (*rho* = 0.560, *p* = 0.006), while anxiety scores demonstrated a negative correlation with SD in the same region (*rho* = –0.530, *p* = 0.024) (Figure 4a, Panel A). These findings suggest that structural alterations in temporal regions may underlie the anxiety symptoms commonly observed in heroin addiction.

#### 3.5.2 Methamphetamine Abstainers (MA)

MA patients exhibited more extensive clinical‐brain correlations. Single‐dose drug use showed significant negative correlations with nodal efficiency in both the CT network (left medial area 38: *rho* = −0.535, *p* = 0.007) (Figure 4c, Panel B) and GI network (right caudal area 7: *rho* = −0.622, *p* = 0.001) (Figure 4d, Panel B). Depression scores showed positive correlations with morphological measures, suggesting complex relationships between mood symptoms and brain structure. Additional marginally significant correlations were observed between drug use frequency, duration of use, and various network efficiency measures (Figure 4d).

Brain diagrams highlight multiple regions including the right caudal area 7 (blue) (Figure 4d, Panel B), right TE 1.0/1.2 (green) (Figure 4b, Panel B), right intermediate lateral area 20 (purple) (Figure 4a, Panel B), and left medial area 38 (yellow) (Figure 4c, Panel B). Depression scores positively correlate with SD in area 20 (*rho* = 0.534, *p* = 0.013) (Figure 4a, Panel B), while drug use duration negatively correlates with CT in TE regions (*rho* = −0.471, *p* = 0.023) (Figure 4b, Panel B). Network analyses reveal a consistent frequency‐dosage dichotomy across both CTNs and GINs: frequency of use positively correlates with nodal efficiency (*E*_i_) in CTNs (*rho* = 0.410, *p* = 0.047) (Figure 4c, Panel B) and GINs (*rho* = 0.524, *p* = 0.009) (Figure 4c, Panel B), while dosage intensity measures show negative correlations with *E*_i_ (dosage per episode in CTNs: *rho* = −0.535, *p* = 0.007 (Figure 4c, Panel B); dosage per episode in GINs: *rho* = −0.622, *p* = 0.001) (Figure 4d, Panel B). This pattern suggests that while frequent methamphetamine use may engage compensatory mechanisms preserving network function, higher dosage intensity consistently impairs brain connectivity across regions and network types. Each scatter plot displays ranked data with Spearman correlations, regression lines, and 95% confidence intervals.

## 4 Discussion

This study provides novel insights into the differential neurobiological mechanisms underlying heroin and methamphetamine addiction through comprehensive morphological brain network analysis. Our findings reveal substance‐specific alterations in brain structure and organization, with heroin abstainers (HA) showing primarily global network disruption and methamphetamine abstainers (MA) exhibiting localized nodal efficiency reductions. Importantly, by studying patients in early abstinence, we demonstrate that these alterations persist beyond acute drug effects, suggesting they represent lasting neurobiological signatures of addiction rather than transient pharmacological states. These distinct patterns suggest different neurotoxic mechanisms and have important implications for understanding addiction pathophysiology and developing targeted therapeutic interventions. While these findings suggest substance‐specific brain alterations, it is important to acknowledge that the significant differences in drug use patterns between heroin and methamphetamine groups (frequency, per‐use dosage, and administration patterns) represent potential confounding factors. The limited sample size precluded robust multivariate analyses to fully disentangle substance‐specific effects from usage‐pattern influences.

### 4.1 Substance‐Specific Morphological Alterations

#### 4.1.1 Cortical Thickness Reductions

The observed cortical thickness reductions in the right temporal areas (TE 1.0/TE 1.2) in both patient groups align with previous neuroimaging studies demonstrating widespread gray matter alterations in substance use disorders (Ersche et al. 2011; Morales et al. 2012). The TE regions are critical components of the superior temporal gyrus, involved in auditory processing, language comprehension, and emotional regulation (Bigler et al. 2007). The bilateral reduction in these regions may contribute to the emotional dysregulation and cognitive deficits commonly observed in addiction patients (Goldstein and Volkow 2011). Twin studies further suggest that genetic factors explain a considerable fraction of inter‐individual variance in cortical morphology (Baaré et al. 2001), implying that inherited vulnerability may interact with drug exposure.

Importantly, the left area 2 showed selective vulnerability in HA patients compared to both MA patients and controls, suggesting heroin‐specific effects on somatosensory processing regions. Area 2 is part of the primary somatosensory cortex and plays a crucial role in tactile discrimination and sensory integration (Kaas 2004). This finding may relate to the altered pain sensitivity and sensory processing abnormalities frequently reported in opioid users (Younger et al. 2008).

#### 4.1.2 Gyrification and Sulcal Morphology Changes

The selective reduction in GI in MA patients, particularly in the left medial area 6 and right dorsomedial parieto‐occipital sulcus, represents a novel finding that may reflect methamphetamine‐specific neurodevelopmental alterations. Gyrification patterns typically establish during early brain development and remain relatively stable throughout life (Zilles et al. 2013). The observed reductions suggest that methamphetamine may interfere with cortical folding patterns, potentially through disruption of white matter integrity or altered cortical connectivity (Luders et al. 2012).

The consistent reduction in SD in the right middle lateral area 20 across both patient groups indicates shared vulnerability of the inferior temporal cortex to substance‐related damage. This region is involved in visual object recognition and semantic processing (Martin 2007), and its alteration may contribute to the cognitive deficits observed in both heroin and methamphetamine users.

#### 4.1.3 Absence of FD Changes

The lack of significant FD changes across groups is noteworthy, as FD measures cortical complexity and folding patterns at a finer scale than traditional morphometric measures (Kiselev et al. 2003). This finding suggests that while substance use disorders affect gross morphological features, they may not significantly alter the fine‐scale complexity of cortical folding patterns. This could indicate that addiction‐related structural changes primarily affect tissue volume and surface area rather than fundamental cortical organization principles.

### 4.2 Network‐Level Alterations: Distinct Pathophysiological Mechanisms

#### 4.2.1 Global Network Disruption in Heroin Abstainers

The increased shortest path length in CT networks of HA patients represents a fundamental alteration in global brain network efficiency. Shortest path length reflects how efficiently information can be transmitted between distant brain regions, with increased values indicating reduced global integration (Bullmore and Sporns 2009). This finding aligns with the concept that opioid addiction involves widespread disruption of brain connectivity networks (Upadhyay et al. 2010).

The global nature of network disruption in HA patients may reflect heroin's broad effects on the opioid receptor system, which is distributed throughout the brain and influences multiple neurotransmitter systems (Koob and Volkow 2016). This widespread disruption could contribute to the severe cognitive impairments, executive dysfunction, and emotional dysregulation observed in heroin users (Baldacchino et al. 2012).

#### 4.2.2 Localized Nodal Efficiency Deficits in Methamphetamine Abstainers

In contrast to the global disruption observed in HA patients, MA patients showed specific reductions in nodal efficiency across multiple networks (CT, GI, and SD networks). The affected regions include the left medial area 38 (temporal pole), right caudal area 7 (superior parietal lobule), and right inferior occipital gyrus. These regions are components of different functional networks involved in semantic processing, spatial attention, and visual processing, respectively (Binder et al. 2009; Corbetta and Shulman 2002).

The localized nature of network alterations in MA patients may reflect methamphetamine's more selective neurotoxic effects, particularly on dopaminergic and serotoninergic systems (Krasnova and Cadet 2009). Methamphetamine's primary mechanism involves blocking dopamine reuptake and reversing dopamine transporter function, leading to excessive dopamine release and subsequent neurotoxicity (Fleckenstein et al. 2007). This mechanism may result in more focal damage to specific brain circuits rather than the widespread network disruption seen with heroin.

### 4.3 Implications of Substance‐Specific Findings

The differential patterns of network alterations provide key insights into substance‐specific neurotoxicity. Heroin, as an opioid agonist, exerts broad effects on the mu‐opioid receptor system distributed throughout the brain, modulating multiple neurotransmitter pathways, including dopamine, GABA, and glutamate. This widespread mechanism likely drives the global network disruption observed in heroin addiction (HA) patients, evidenced by increased shortest path length in cortical thickness (CT) networks. This disruption potentially causes pervasive cognitive impairments through diffuse synaptic pruning and reduced inter‐regional connectivity (Volkow et al. 2019).

Conversely, methamphetamine acts as a dopamine reuptake inhibitor and releaser, inducing selective neurotoxicity in dopaminergic circuits, particularly affecting fronto‐striatal and temporo‐parietal regions (Krasnova and Cadet 2009). This mechanism explains the localized nodal efficiency deficits in methamphetamine addiction (MA) patients across CT, GI, and SD networks, affecting key hubs like the temporal pole (emotional processing) and superior parietal lobule (visuospatial attention). These findings suggest different treatment approaches may be optimal for each addiction type, with heroin addiction potentially benefiting from holistic interventions like broad‐spectrum neuromodulation, while methamphetamine addiction could respond better to circuit‐specific approaches such as targeted cognitive training.

### 4.4 Persistence Versus Recovery in Early Abstinence

Our findings demonstrate that several key alterations persist during early abstinence, providing direct evidence that these represent enduring addiction‐related brain changes rather than temporary drug states. The MA‐specific reductions in GI (left medial area 6; right dorsomedial parieto‐occipital sulcus) are particularly compelling given that gyrification patterns are largely established during development and remain relatively stable across the lifespan, making short‐term state explanations unlikely.

Similarly, the global network disorganization in heroin users (increased shortest path length) and localized nodal efficiency disruptions in methamphetamine users that we observed during abstinence align with broader network‐level vulnerabilities that extend beyond cessation. The dose‐dependent associations we found (higher methamphetamine dosage linked to lower nodal efficiency) further support interpretations of residual neurotoxic burden rather than acute effects. However, the positive correlations between usage frequency and certain morphological measures may indicate early compensatory reorganization processes beginning during abstinence.

### 4.5 Clinical Correlations and Functional Implications

#### 4.5.1 Abstainers: Morphology‐Behavior Relationships

The significant positive correlation between drug abuse frequency and SD in the right middle lateral area 20 suggests that more frequent heroin use is associated with greater morphological alterations in visual association areas. This relationship may reflect cumulative neurotoxic effects, where repeated exposure leads to progressive structural damage (Bolla et al. 2003).

The negative correlation between anxiety scores and SD in the same region provides insights into the emotional consequences of structural brain alterations. This finding suggests that morphological changes in temporal regions may contribute to the anxiety and emotional dysregulation commonly observed in addiction (Koob and Volkow 2010).

#### 4.5.2 Methamphetamine Abstainers: Dose‐Dependent Network Effects

The negative correlations between single‐dose drug use and nodal efficiency in both CT and GI networks highlight the dose‐dependent effects of methamphetamine on brain network organization. Higher doses appear to cause greater disruption of local network efficiency, which may contribute to the cognitive deficits observed in chronic methamphetamine users (Scott et al. 2007).

The more extensive clinical‐brain correlations observed in MA patients compared to HA patients suggest that methamphetamine's effects on brain structure may be more directly related to usage patterns and clinical symptomatology. This finding supports the concept that methamphetamine addiction involves more complex relationships between drug exposure parameters and neurobiological outcomes.

### 4.6 Neurobiological Mechanisms and Therapeutic Implications

The differential patterns of brain alterations between heroin and methamphetamine users have important implications for understanding addiction pathophysiology and developing targeted treatments. The global network disruption in heroin users suggests that therapeutic interventions should focus on restoring widespread connectivity and integration across brain networks. This might involve approaches that target multiple neurotransmitter systems or utilize network‐based neuromodulation techniques.

In contrast, the localized nodal efficiency deficits in methamphetamine users suggest that more targeted interventions focusing on specific brain circuits may be more effective. Cognitive rehabilitation programs targeting the affected domains (e.g., attention, visual processing, semantic memory) might be particularly beneficial for this population.

### 4.7 Study Limitations and Future Directions

Several limitations should be acknowledged. First, our sample was predominantly male, which limits generalizability to female patients. While this distribution reflects typical clinical populations seeking treatment for these disorders, future studies should include larger, gender‐balanced samples to examine potential sex‐specific effects. Second, the cross‐sectional design prevents determination of causality and does not capture the dynamic nature of brain recovery during abstinence. Importantly, our cross‐sectional design cannot dissociate residual damage from recovery‐related remodeling or premorbid vulnerability, emphasizing the need for longitudinal trajectories across varying abstinence windows. The clinical implications of our findings should be interpreted cautiously given the potential confounding effects of different usage patterns between substance groups. While the observed differences align with known pharmacological distinctions between heroin and methamphetamine, definitive clinical recommendations require replication in larger studies with adequate statistical power to control for usage‐related variables.

The absence of fractal‐dimension alterations warrants higher‐resolution morphological metrics, such as the multilevel descriptors proposed by Lv et al. (2021), which may reveal subtler geometric differences. Additionally, the relatively short abstinence period (less than six months) may not capture the full extent of structural recovery that can occur with prolonged abstinence (Connolly et al. 2013).

Future studies should include larger, gender‐balanced samples and longitudinal designs to track brain changes during abstinence and recovery. Integration of functional neuroimaging data with morphological measures could provide more comprehensive insights into the relationship between structural alterations and functional impairments.

## 5 Conclusion

This study demonstrates that heroin and methamphetamine exert distinct effects on brain morphology and network organization, with heroin primarily causing global network disruption and methamphetamine leading to localized nodal efficiency deficits. By studying patients in early abstinence, we show that these alterations persist beyond acute drug effects, representing lasting neurobiological signatures of addiction. The observed associations between morphological alterations and clinical symptoms—including anxiety in heroin users and depression in methamphetamine users—highlight the functional significance of these structural brain changes. These findings advance our understanding of substance‐specific neurobiological mechanisms and suggest that different therapeutic approaches may be optimal for different types of addiction. The observed morphological alterations and their clinical correlations provide valuable insights into the neural basis of addiction‐related cognitive and emotional impairments, potentially informing the development of more targeted and effective treatment strategies.

## Author Contributions

Study conceptualization: XLZ, WBL, JPZ; Data curation: XLZ, WBL; Formal analysis: JPZ, MWL; Clinical investigation: XLZ, MWL; Supervision: MWL; Writing – original draft: XLZ, WBL; Writing – review & editing: JPZ, MWL.

## Funding

This work was supported by the National Natural Science Foundation of China (No. 81471661).

## Ethics Statement

This study was conducted in accordance with the Declaration of Helsinki and was approved by the Ethics Committee of Longgang Central Hospital. Ethics approval was obtained from the institutional review board.

## Consent

All participants provided informed consent before participation.

## Conflicts of Interest

The authors declare no conflicts of interest.

## Peer Review

The peer review history for this article is available at https://doi.org/10.1002/brb3.71051.

## Data Availability

All data supporting the findings of this study are available from the corresponding author upon reasonable request.
